# Prevalence and associated factors of Leukemia in Africa: A systematic review and meta-analysis

**DOI:** 10.1371/journal.pone.0354814

**Published:** 2026-08-03

**Authors:** Solomon Gedfie, Melese Abate Reta, Muluken Gashaw, Alembante Bazezew, Abdu Jemal, Nigatu Getu, Mengistu Tesfa Alula, Bewuketu Belete Alemu, Marye Nigatie, Getinet Kumie, Zewdu Tefera, Brhanu Kassanew, Ermias Getachew, Assefa Sisay, Yalewayker Gashaw, Mesfine Desale, Ephrem Tamrat, Zelalem Dejazmach, Wagaw Abebe, Agenagnew Ashagre, Tadesse Misganaw, Woldeteklehaymanot Kassahun

**Affiliations:** 1 Department of Medical Laboratory Sciences, College of Health Sciences, Woldia University, Woldia, Ethiopia; 2 Department of Medical Microbiology, Faculty of Health Sciences, University of Pretoria, Pretoria, South Africa; 3 Woldia Cmprehensive Specialized Hospital, Woldia, Ethiopia; Tekirdag Namik Kemal University: Tekirdag Namik Kemal Universitesi, TÜRKIYE

## Abstract

**Background:**

Leukemia is a group of diverse and biologically distinct blood cancers. While progress has been made in combating infectious diseases, chronic diseases like leukemia are increasingly prevalent. Current data on leukemia’s prevalence in Africa are inconsistent, necessitating a comprehensive systematic review. This study aims to determine the pooled prevalence and associated risk factors of leukemia across Africa through a systematic review and meta-analysis.

**Method:**

This systematic review and meta-analysis were conducted based on the Preferred Reporting Items for Systematic Reviews and Meta-Analyses (PRISMA) guidelines. A comprehensive literature search was conducted across multiple databases, including PubMed/MEDLINE, Scopus, and Science Direct, supplemented by searches using the Google Scholar and manual searches to identify eligible studies. Statistical analysis was performed using STATA version 11, with a random-effects model employed to compute pooled estimates. Higgen’s I^2^ test statistics and meta-regression was conducted to explore potential sources of heterogeneity. Publication bias was assessed visually by Funnel plots and statistically using Egger’s weighted regression test, a p-value of less than 0.05 indicates the presence of significant publication bias.

**Results:**

A total of 17,546 articles were retrieved, of which fifteen studies, which recruited 42,884 individuals, were included in the meta-analysis. The pooled prevalence of leukemia was 5.09% (95% CI: 4.02, 6.17) with I^2^ of 97.1%. Due to the heterogeneity, subgroup analyses by country showed that the highest prevalence was 10.58% in Nigeria, and the lowest was 0.58% in Zambia. Subgroup analysis based on publication year revealed 4.16% among studies conducted before 2015 and 6.67% among studies conducted after 2016. Additionally subgroup by sample size showed: smaller sample sizes (<384 participants) yielded a higher pooled estimate of 7.99%, conversely, studies with larger sample sizes (≥385 participants) showed 3.61%. Whereas subgroup analysis by population showed that 3.38% in children and 7.15% in the general population. Among the factors identified, rural residency (OR: 56.98, 95% CI: 9.97–326.70) and older age (OR: 3.90, 95% CI: 1.39–10.98) were significant predictors of leukemia in Africa.

**Conclusion:**

This review reveals a pooled prevalence of 5.09%, with rural residency and older age identified as significant predictors of leukemia in Africa, underscoring a public health concern.

## Introduction

Leukemia is a diverse group of blood cancers that originate in the bone marrow and other blood-forming tissues [1]. These cancers involve the unchecked growth of immature blood cells, interfering with normal blood cell production and potentially spreading to other organs [2]. Leukemia is classified according to both cell lineage and how quickly the disease progresses. The cell lineage determines whether it is lymphoid or myeloid leukemia, while the progression rate categorizes it as either acute or chronic [2,3]. This essential classification system informs how clinicians diagnose, treat, and predict outcomes for patients. When categorized clinically by disease progression (acute or chronic) and cell lineage (myeloid or lymphoid), this blood cancer exhibits unique biological characteristics across its various subtypes [4].

Leukemia represents a major global health challenge, affecting millions of people around the world. Its incidence, prevalence, and death rates differ considerably depending on geographic region and population group. Worldwide, the number of new leukemia diagnoses rose from 311,648 in 1990–461,423 in 2021, and projections indicate this figure will exceed half a million by 2050 [5]. Among the new cases recorded in 2021, 57.2% occurred in males, 70.8% involved individuals aged 45 or older (middle-aged and elderly), and acute myeloid leukemia (AML) remained the predominant subtype, accounting for 31.4% of cases [5–8]. Between 1990 and 2021, leukemia incidence fell notably from 6.9 to 5.6 per 100,000, mortality dropped from 5.6 to 3.9, and disability-adjusted life years (DALYs) decreased from 266.3 to 136.9 [5].

In Africa, especially Sub-Saharan Africa, the impact of leukemia poses distinct difficulties. Although accurate statistics are hard to obtain because of poor cancer registration and data collection systems, existing evidence points to a major gap in leukemia incidence, prevalence, and death rates when compared with high-income nations [6,9]. In 2021, East Africa experienced the highest leukemia burden, reporting 8,286 adult cases (age-standardized incidence rate [ASIR] of 4.5) and 2,954 pediatric cases (ASIR of 1.6). In contrast, West Africa had the lowest burden, with 3,612 adult cases (ASIR 2.3) and 790 pediatric cases (ASIR 0.47). Middle Africa and Southern Africa recorded moderate burdens: adults in Middle Africa accounted for 2,196 cases (ASIR 3.1), while those in Southern Africa accounted for 1,981 cases (ASIR 5.0) [10].

While global efforts have successfully lessened the toll of infectious diseases over the past several decades, chronic non-communicable conditions like leukemia are becoming an increasing public health threat, as their prevalence continues to rise steadily across the globe [11]. The demographic patterns of blood cancers in Africa are still not well defined, and it remains unclear whether they mirror those seen in high-income nations. Recent data from South Africa uncovered a notable disparity: White children had three times the incidence of pediatric hematologic malignancies compared to Black children. This observation is especially significant within the African setting, where population categories frequently align closely with social and economic gradients [12].

In developing countries, cancer plays a major role in both mortality and disability, as healthcare systems frequently lack the necessary resources to combat the disease effectively. Africa, in particular, is experiencing a rapid increase in its cancer burden. By 2030, projections suggest over one million new cancer cases and nearly 800,000 deaths, representing an 85% rise compared to 2008 levels [13].

The etiology of leukemia and its subtypes remains incompletely understood, partly because of the variety of abnormalities and the numerous potential risk factors. Genetic syndromes like Down syndrome, Li-Fraumeni syndrome, and neurofibromatosis have been connected to a predisposition for leukemia in young adults [14]. Benzene exposure is unequivocally associated with a heightened risk of acute myeloid leukemia (AML). Ionizing radiation has been linked to acute lymphoblastic leukemia (ALL), especially in children, as well as to AML and chronic myeloid leukemia (CML). Furthermore, viral infections have been implicated in lymphoproliferative disorders, with Epstein-Barr virus (EBV) and human T-cell lymphotropic virus type-1 (HTLV-1) playing well-documented roles in specific lymphoid malignancies [14–16].

The exact cause of leukemia is not fully understood, but it is believed to result from a combination of genetic predisposition and environmental influences [17]. Established risk factors include tobacco smoking, exposure to ionizing radiation, certain chemical agents (such as benzene), prior chemotherapy or radiation therapy, and genetic disorders like Down syndrome [18]. Additionally, individuals with a family history of leukemia or certain lifestyle factors may also face an increased risk of developing the disease [19]. Although progress has been made in understanding the molecular and genetic mechanisms underlying leukemia, data on its burden in Africa remain inconsistent. Consequently, this systematic review and meta-analysis aims to establish the pooled prevalence of leukemia in Africa and identify associated factors.

## Methods

### Design and protocol registration

This systematic review and meta-analysis was conducted based on Preferred Reporting Items for Systematic Review and Meta-Analysis (PRISMA) protocols [20]. The protocol has been registered with the International Prospective Register of Systematic Reviews (PROSPERO) under registration number CRD420251017790.

### Search strategy

A comprehensive literature search was conducted across multiple electronic databases, including Scopus, PubMed/MEDLINE, Science Direct, and Google Scholar, along with relevant institutional repositories. The search was performed independently by two reviewers (SG and MAR). The search included all available publication dates and was restricted to English-language articles published up to March 30, 2025. A systematic search strategy was employed, utilizing both individual and combined search terms linked by Boolean operators (“AND,” “OR”). The primary search terms comprised “leukemia,” “hematological malignancy,” “blood cancer,” “prevalence,” “incidence,” “epidemiology,” and “Africa,” as well as related terms such as “magnitude” and “blood disorders.”

The search string used in the PubMed was:-(((((((((((Prevalence) OR (Magnitude)) OR (Incidence)) AND (Predictors)) OR (factors)) OR (Risk-Factors)) OR (Associated factors)) AND (Leukemia)) OR (Hematological Malignancy)) OR (Cancer)) AND (Africa) by adding African search filter. Additional studies were searched by manual search and by looking into references in pertinent papers. The search strategy and number of articles retrieved from the searched databases and additional searches are depicted in the additional file (S1 Table in S1 File).

### Eligibility criteria

This systematic review incorporated peer-reviewed published journal articles, as well as studies available through institutional electronic repositories, that reported on leukemia prevalence and associated factors. Eligible study designs included case-control studies, cohort studies (both prospective and retrospective), and cross-sectional studies that measured the relevant outcome variables. Eligible studies were limited to English-language articles published up to March 30, 2025. The review excluded case reports, case series, narrative reviews, editorials, and studies available only as conference abstracts. Agreements on the inclusion and exclusion of the articles were held through the involvement of authors (MN, GK, ZT and MTA).

### Study selection and quality assessment

To organize search results and remove duplicate studies, all retrieved articles were imported into EndNote X21 (Thomson Reuters, New York, USA). The literature search was conducted across electronic databases, trial registers, and Google Scholar by three reviewers (SG, WTK, and ZT). Title screening was performed independently by two reviewers (SG, AJ). Abstract screening was carried out independently by three reviewers (MN, AA, and BK). Full-text screening was conducted independently by four reviewers (SG, AB, BM, and MG). Any disagreements arising between reviewers at any stage were resolved through discussion and consultation with additional reviewers (WA, BBA, and AS). Additionally, the methodological quality of included studies was appraised using the Joanna Briggs Institute (JBI) critical appraisal tools, by reviewers (MAR, TM, GK, and YG) [21].

The quality appraisal guideline contains 10 evaluation domains or categories to evaluate the internal and external validity. The items are: 1. (a) representativeness for the population, (b) sampling frame, (c) Ways of study unit selection, (d) bias due to non-response, (e) data source (primary data), (f) acceptability of case definition, (g) reliability and validity of study tool, (h) mode of data collection, (i) appropriateness of numerator and denominator, and (j) summary. Each category was evaluated as low or high risk of bias. Unclear was considered as a high risk of bias. Eventually, the summary risk of bias was determined according to the number of the high risk of bias per study: that is defined as low (0–3), moderate [4–6], and high [7–9] depicted quality score (S2 Table in S1 File).

### Data extraction

Data extraction was accomplished by two reviewers (SG and MN) from studies that fulfill the eligibility criteria. The extracted data were summarized into an MS Excel spreadsheet. Disagreements were resolved through consensus and discourse with other reviewers (MAR, MN, EG and ET). The following data were systematically extracted from each included study for analysis: first author’s name, publication year, study design, and country where the research was conducted. Additionally, we collected data on study population characteristics, including total number of participants and their age distribution. Key outcome measures extracted were the number of confirmed leukemia cases and calculated prevalence rates. Additionally, to analyze factors associated with leukemia, we extracted factors that were reported as statistically significant in the primary studies. Factors that were not significant or not reported were not included in this analysis. We also documented all significant factors associated with leukemia that were reported in the studies. This comprehensive data extraction approach enabled thorough evaluation and synthesis of the available evidence on leukemia epidemiology across African populations (Table 1).

**Table 1:** Characteristics of included studies in the meta-analysis of prevalence of leukemia in Africa.

Author	Year of publication	country	Study design	population	Sample size	Number of cases	Mean age	Leukemia prevalence (%)	Quality score
Endalamaw A, *et al* [25]	2019	Ethiopia	prospective cross-sectional	children	1257	2	32.4 month	0.8	8
Mousay HY*, et al* [33]	2014	Libya	Retrospective cross-sectional	children	182	5	8.3 ± 1	2.5	8
Haouas H,*et al* [34]	2003	Tunisia	Retrospective cross-sectional	General population	1007	42	1–90yrs	4.2	8
Abuidres DO,*et al* [38]	2015	Sudan	Retrospective cross-sectional	children	15387	340	7 ± 5	2.2	8
Kassahun W*,et al* [26]	2019	Ethiopia	prospective cross-sectional	General population	332	31	23 ± 16.5	9.3	8
Ugwu NI*, et al* [30]	2020	Nigeria	Retrospective cross-sectional	Adult	135	7	49 ± 17	5.2	8
Hussein S*, et al* [35]	2024	Egypt	case-control	General population	1137	0	50 ± 16.5	Factors reported	8
Enawgaw B,*et al* [27]	2019	Ethiopia	Retrospective cross-sectional	General population	1342	96	41.49 ± 16.3	7.1	8
Maybin Kalubula, *et al* [36]	2014	Zambia	retrospective observational	General population	21512	126	undefined	0.585	8
Robert N, *et al* [37]	1993	Rwanda	Retrospective cross-sectional	General population	293	6	undefined	2.05	8
Ebrahim H, *et al* [28]	2021	Ethiopia	prospective cross-sectional	General population	228	26	35.51 ± 14.02	1.14	8
Alamin AA, *et al* [39]	2017	Eritrea	Retrospective cross-sectional	General population	204	20	undefined	9.8	8
Kagu M, *et al* [31]	2011	Nigeria	Retrospective cross-sectional	General population	236	40	undefined	16.95	8
Woldu M, *et al* [29]	2016	Ethiopia	prospective cross-sectional	General population	142	13	42.27 ± 16.8	9.09	8
Babatunde T, *et al* [32]	2010	Nigeria	Retrospective cross-sectional	Children	625	64	<15 years	10.2	8

### Statistical analysis

The data were checked for completeness in Microsoft Excel before being transferred to STATA version 11 for final analysis. The effect size was estimated using a random-effects model, presented with a 95% confidence interval (95% CI) [22]. Forest plots were generated to display the overall pooled prevalence, along with the relative weight assigned to each included study. The degree of heterogeneity was assessed using Higgins’ I^2^ statistic, with I^2^ values categorized as low (25%), moderate (50%), and high (75%) [23]. To explore potential sources of heterogeneity, sub-group analyses were performed based on the country where the primary study was conducted, year of publication, study population, and sample size. Furthermore, meta-regression analysis was conducted to investigate possible sources of heterogeneity. A sensitivity analysis was conducted by sequentially excluding individual studies to determine whether any single study significantly influenced the pooled estimate. To assess potential publication bias, funnel plots were visually inspected, and Egger’s weighted regression test was performed. In Egger’s test, a p-value of less than 0.05 was considered indicative of statistically significant publication bias [24].

### Ethics approval and consent to participate

As this study is a systematic review and meta-analysis of previously published data, it did not involve direct patient contact or primary data collection. Therefore, ethical approval and informed consent were not required.

## Results

### Searching results

**Fig 1:**
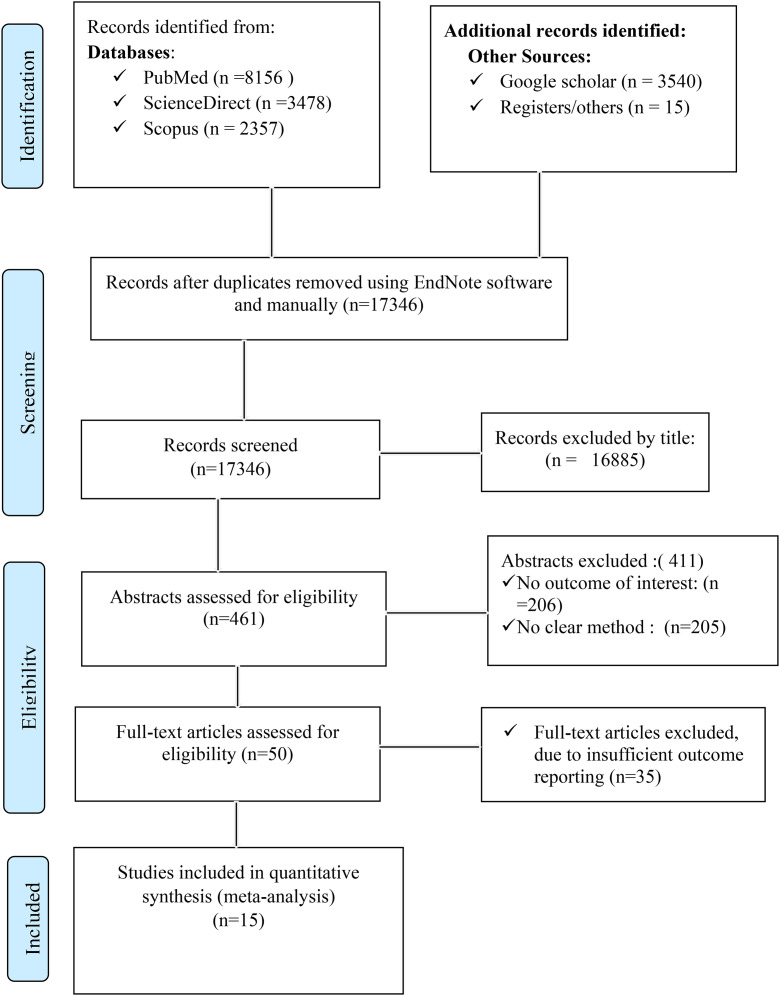
PRISMA flow diagram showing the results of the search and reasons for exclusion on systematic review and meta-analysis of leukemia in Africa [20].

This systematic review and meta-analysis included published and unpublished articles on the prevalence and predictors of leukemia in Africa. The search strategy employed the following databases: PubMed/MEDLINE, ScienceDirect, Scopus, and Google Scholar. The initial search identified 17,546 studies. After removing 200 duplicate records, 17,296 studies were excluded during title and abstract screening. Following the screening of 50 full-text articles for eligibility, 35 were excluded because they did not report the relevant outcome variable. Consequently, 15 studies met all inclusion criteria and were included in the final meta-analysis (Fig 1).

### Characteristics of included studies

A total of fifteen studies met the inclusion criteria for this systematic review and meta-analysis. Among the included studies: five were conducted in Ethiopia [25–29], three were in Nigeria [30–32] and the others were conducted in Libya [33], Tunisia [34], Egypt [35], Zambia [36], Rwanda [37], Sudan [38] and Eritrea [39]. The sample size ranges from 135 in Nigeria [30] to 21512 in Zambia [36]. The highest prevalence of leukemia (16.95%) was reported in Nigeria [40], whereas the lowest (0.585%) was found in Zambia [36] (Table 1).

### Publication bias

**Fig 2:**
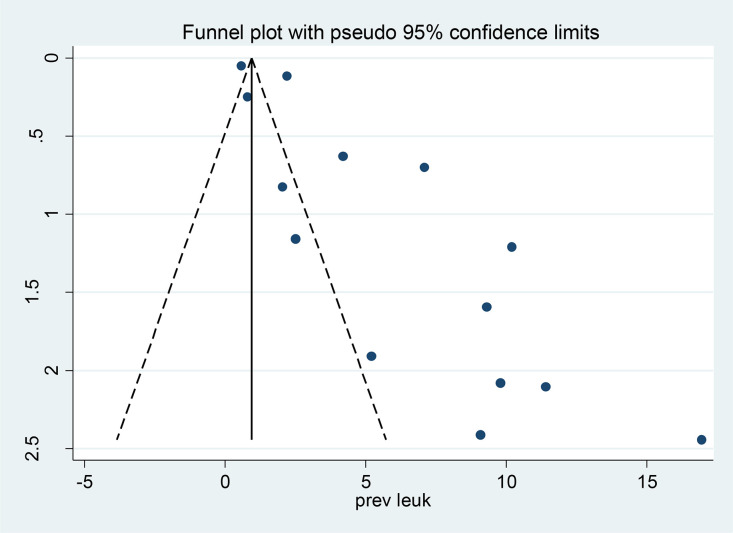
Funnel plot on the prevalence of leukemia in Africa.

Publication bias occurs when studies with statistically significant or favorable results are more likely to be published than those with low prevalence findings. Included studies were assessed for potential publication bias visually by funnel plot and egger’s test statistics. The funnel plot (Fig 2) is asymmetric, shows an evidence of publication bias. The egger’s weighted regression statistics showed that (p < 0.05) (in this case P = 0.001), also showed presence of publication bias (Table 2). This suggests the necessity of performing a trim-and-fill analysis to assess and adjust for potential publication bias in the dataset.

**Table 2:** Egger’s test for prevalence of leukemia in Africa.

Std_Eff	Coef.	Std. Err.	t	P > t	[95% Conf. Interval]	
slope	.4887655	.2046046	2.39	0.034	.0429705	.9345605
bias	5.175992	1.180609	4.38	0.001	2.603666	7.748318

CI: Confidence interval; Std Eff: Standard effect; Coef: coefficient; Std. Err: Standard error.

### Trim and fill analysis of pooled estimate of leukemia in Africa

**Fig 3:**
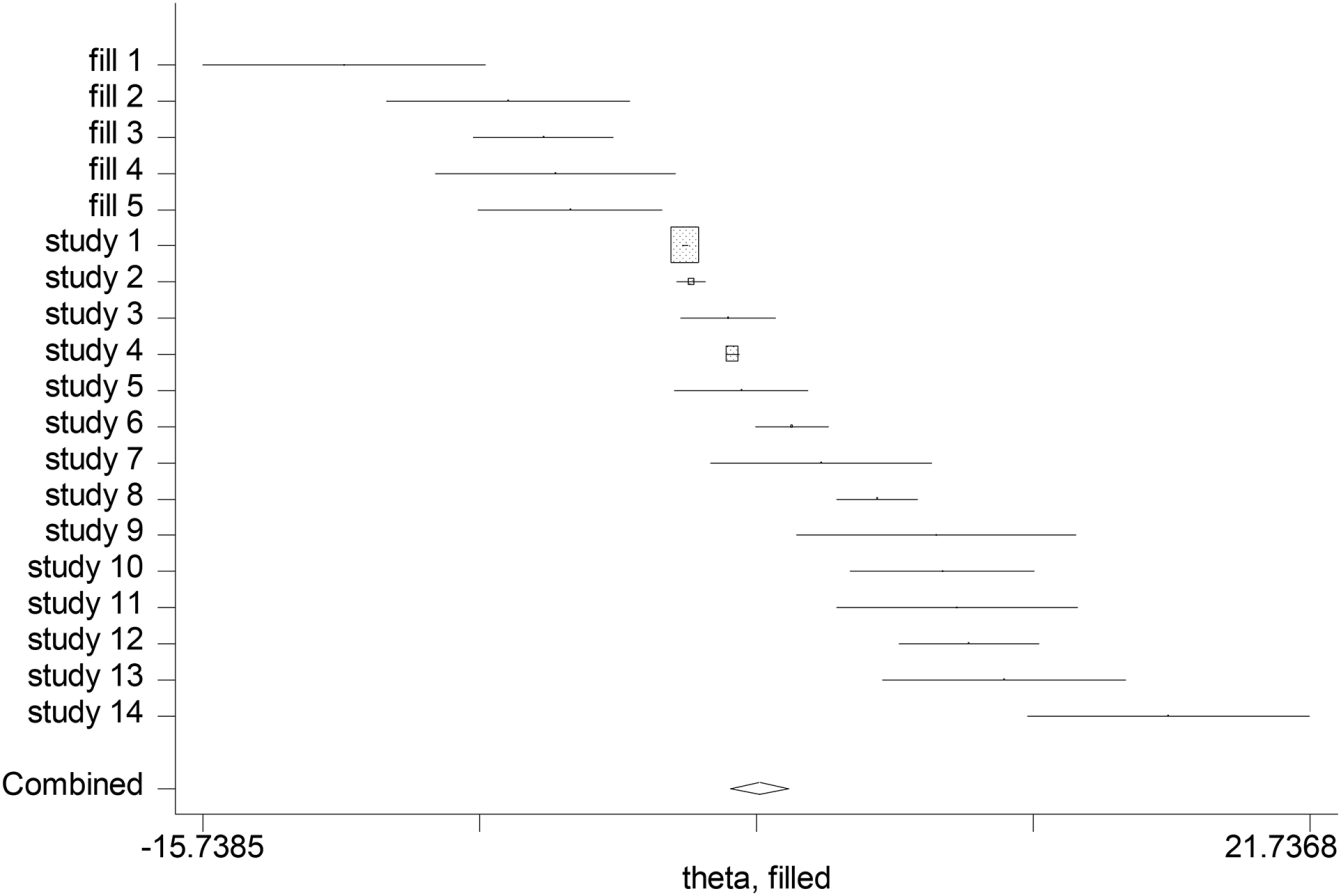
Trim and fill analysis of the pooled prevalence of leukemia in Africa.

Due to the presence of publication bias (*p-value* = 0.001) and evidence of asymmetry from the funnel plot, so, nonparametric trim and fill analysis was performed. The trim-and-fill method imputed five additional studies to address potential publication bias. Based on the random-effects model, after trim and fill analyses the adjusted pooled prevalence of leukemia in Africa changed from 5.09% (95% CI: 4.02, 6.17) to 3.102% (95% CI: 2.104–4.099). This suggests that studies with smaller effects, which were either excluded or failed to be published, were incorporated into the model using the trim-and-fill method. As a result, the pooled estimate was lower compared to the original analysis conducted without trim-and-fill adjustment (Fig 3).

### Sensitivity analysis

A sensitivity analysis was conducted using a random-effects model to assess the influence of individual studies on the overall pooled estimate of leukemia prevalence in Africa. The analysis involved systematically omitting one study at a time. The results indicated that no single study had a substantial effect on the pooled estimate (Table 3).

**Table 3:** Sensitivity analysis of included studies on the prevalence of leukemia in Africa.

Study omitted	Point | Estimate	[95% Conf. Interval]	
		Lower CI	Upper CI
Endalamaw A 2019)	5.74	4.52	6.95
Mousay HY (2024)	5.30	4.19	6.42
Haouas H (2003)	5.16	4.04	6.27
Abuidres DO (2015)	6.05	4.46	7.63
Kassahun W (2019)	4.80	3.71	5.88
Ugwu NI (2020)	5.08	3.99	6.18
Enawgaw B (2019)	4.74	3.68	5.79
Maybin K (2014)	6.10	4.63	7.58
Robert N (1993)	5.40	4.27	6.52
Ebrahim H (2021)	4.78	3.71	5.85
Alamin AA (2017)	4.86	3.78	5.94
Kagu M (2011)	4.61	3.56	5.66
Woldu M (2016)	4.93	3.85	6.01
Babatunde T (2010)	4.59	3.53	5.65
Combined	5.09	4.02	6.17

### Prevalence of leukemia in Africa

**Fig 4:**
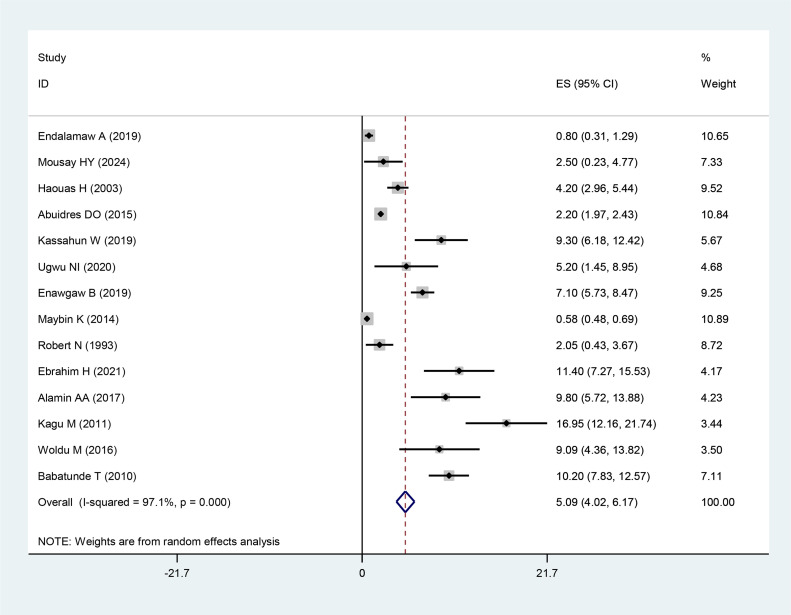
Forest plot showing prevalence of leukemia in Africa.

Due to substantial between-study heterogeneity (I^2^ = 97.1%, p < 0.001), we employed a DerSimonian-Laird random-effects model to estimate the overall prevalence. Under this model, the pooled prevalence of leukemia in Africa was 5.09% (95% CI: 4.02–6.17) (Fig 4).

**Fig 5:**
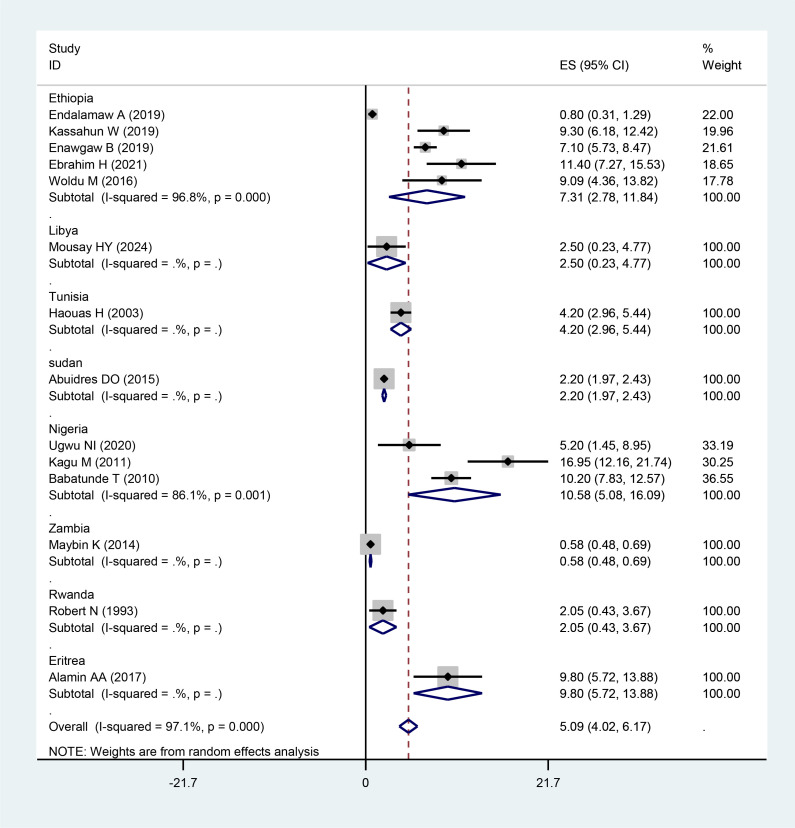
Forest plot showing sub-group analysis of the pooled prevalence of leukemia in Africa by Country.

**Fig 6:**
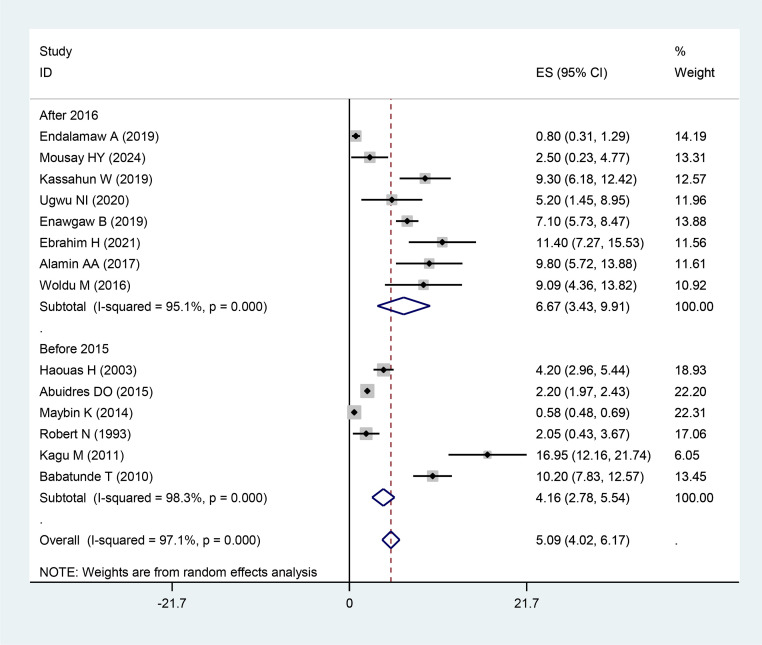
Forest plot showing sub-group analysis of the pooled prevalence of leukemia in Africa by year of publication.

**Fig 7:**
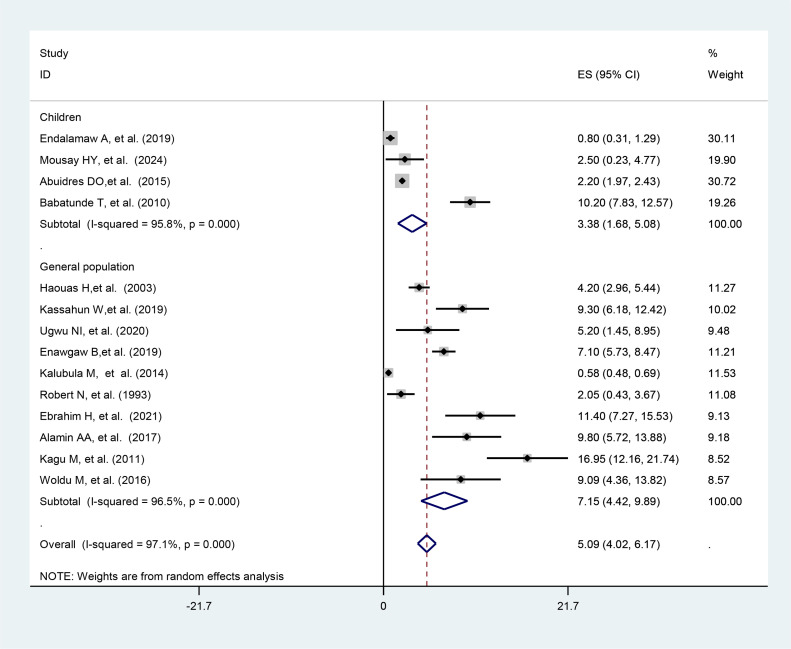
Forest plot showing sub-group analysis of the pooled prevalence of leukemia in Africa by study population.

**Fig 8:**
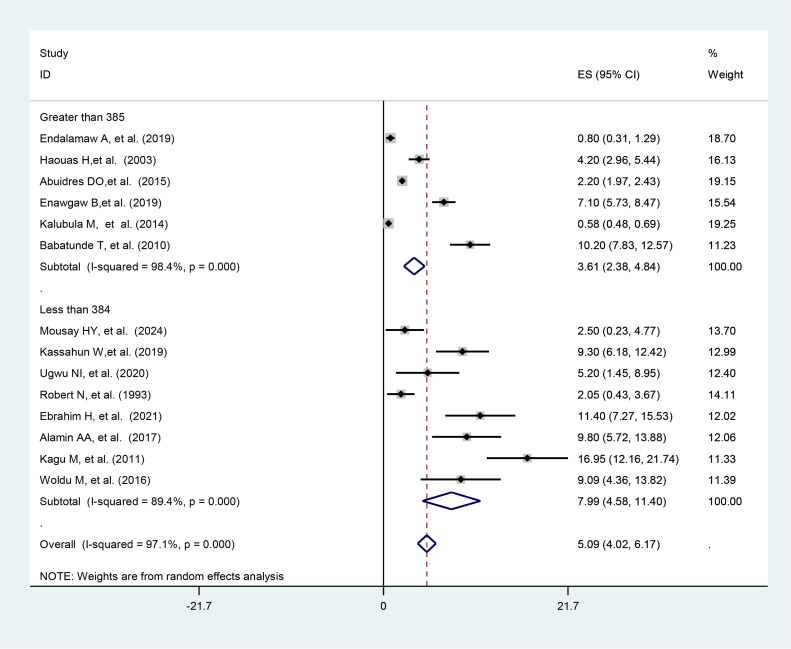
Forest plot showing sub-group analysis of the pooled prevalence of leukemia in Africa by sample size range.

Given the significant heterogeneity observed across the included studies (I^2^ = 97.1%, p < 0.001) the pooled prevalence should be interpreted with caution due to extreme heterogeneity (I^2^ = 97.1%) and evidence of publication bias (Egger’s test p = 0.001) under such conditions, the pooled estimate may not represent a single meaningful value. To explore potential sources of the substantial heterogeneity, we conducted subgroup analyses based on country of study, publication year, study population, and sample size. These analyses revealed significant geographic variation in the pooled prevalence of leukemia across Africa. Nigeria reported the highest estimate at 10.58% (95% CI: 5.08–16.09), followed by Ethiopia at 7.31% (95% CI: 2.78–11.84), while Zambia recorded the lowest prevalence at 0.58% (95% CI: 0.48–0.69) (Fig 5). When stratified by publication year, the pooled prevalence of leukemia was 4.16% (95% CI: 2.78–5.54) for studies published before 2015, with substantial heterogeneity (I^2^ = 98.3%, p < 0.001). In contrast, studies published in 2016 or later yielded a higher pooled estimate of 6.67% (95% CI: 3.43–9.91), although considerable heterogeneity persisted (I^2^ = 95.1%, p < 0.001) (Fig 6). Subgroup analysis by study population showed a pooled prevalence of 3.38% (95% CI: 1.68–5.08) among children, compared with 7.15% (95% CI: 4.42–9.89) in the general population, suggesting a significantly higher burden in the latter group. Both subgroups exhibited substantial heterogeneity (I^2^ > 95%, p < 0.001), indicating considerable variability across individual studies. These observed differences may be attributable to variations in risk factor profiles, diagnostic methodologies, or sampling strategies between populations (Fig 7). Subgroup analysis by sample size revealed notable variations in the pooled prevalence of leukemia. Studies with smaller sample sizes (<384 participants) yielded a higher pooled estimate of 7.99% (95% CI: 4.58–11.40), albeit with considerable heterogeneity (I² = 98.4%, p < 0.001). In contrast, studies with larger sample sizes (≥385 participants) reported a lower pooled prevalence of 3.61% (95% CI: 2.38–4.84), accompanied by moderate heterogeneity (I² = 89.4%, p < 0.001) (Fig 8).

### Meta regression

A meta-regression analysis was carried out to explore potential sources of heterogeneity in leukemia prevalence within Africa, focusing on study year and sample size as covariates. Results indicated that neither factor significantly accounted for the observed variability (Table 4).

**Table 4:** Meta-regression analysis for included studies to assess the sources of heterogeneity.

Prev.leukemia	Coef.	Std. Err.	t	P > |t|	[95%Conf.Interval]	
studyyear	.072843	.1481499	0.49	0.633	−.2532327	.3989188
samplesize	−.0003261	.0001778	−1.83	0.094	−.0007174	.0000653
_cons	−139.4748	298.3941	−0.47	0.649	−796.2357	517.2861

### Factors associated with leukemia prevalence

**Fig 9:**
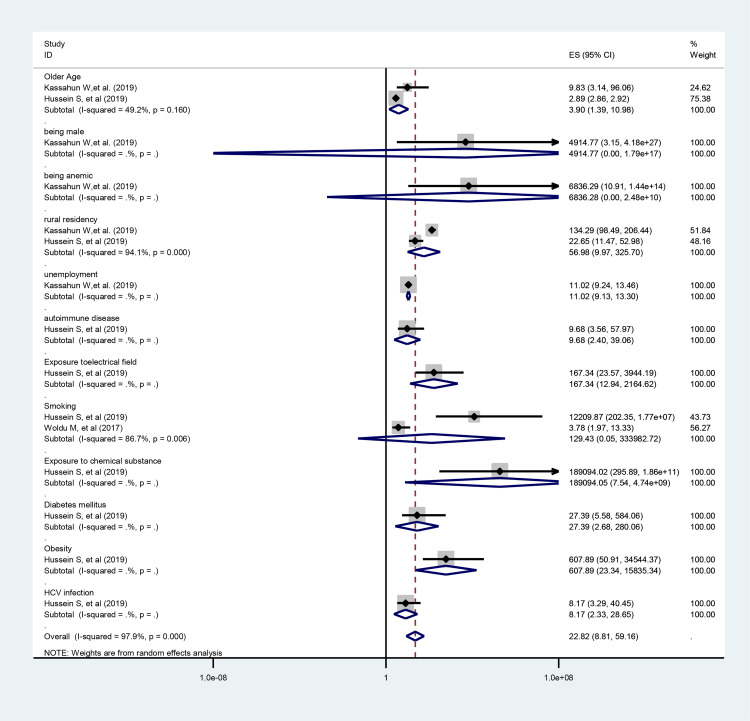
Forest plot showing factors associated with leukemia prevalence in Africa.

The meta-analysis evaluated multiple potential risk factors for leukemia, including older age, anemia, male sex, smoking status, rural residency, unemployment, autoimmune diseases, hepatitis C virus (HCV) infection, diabetes mellitus, electrical field exposure, chemical substance exposure, and obesity. Among these, older age and rural residency emerged as significant predictors of leukemia in the African population. Pooled analysis revealed that individuals residing in rural areas were 56.98 times more likely to develop leukemia than urban residents (OR: 56.98, 95% CI: 9.97–326.70). Similarly, older age was associated with a nearly 4-fold increased risk (OR: 3.90, 95% CI: 1.39–10.98) (Fig 9). Risk factors reported by only a single study were not eligible for pooled analysis. Instead, these factors are presented descriptively as individual study-level findings. To be included in the meta-analysis and reported as a summary effect, a risk factor had to be examined in at least two independent studies. Therefore, the forest plot presented below should be interpreted with caution.

## Discussion

The present systematic review and meta-analysis was conducted to estimate the pooled prevalence of leukemia and to explore its associated risk factors in Africa, drawing on data from 15 eligible studies. Using a random-effects model, the pooled prevalence of leukemia in Africa was 5.09% (95% CI: 4.02–6.17), with substantial heterogeneity across studies (I^2^ = 97.1%, p < 0.001). Given this considerable heterogeneity, the pooled estimate should be interpreted with caution. The findings of the current systematic review and meta-analysis is in agreement with studies conducted in Nigeria 5.2% [30], and Tunisia 4.2% [34]. On the contrary the findings of the current systematic review and meta-analysis reports higher pooled prevalence as compared to studies conducted in Ethiopia 0.8% [25], Libya 2.5% [33], Sudan 2.2% [38], Zambia 0.585% [36] and Rwanda 2.05% [37]. The wide variation in prevalence estimates likely reflects differences in sample size, population characteristics, geographic setting, sociodemographic factors, specimen type, and diagnostic approaches.

On the other hand, the pooled prevalence reported in the current systematic review and meta-analysis is lower than that reported by Mulatie Z et al. (53.69%) [41], as well as individual studies conducted in Pakistan [42], Bangladesh [43], and India [44]. The observed discrepancies in these findings may be attributed to several factors, including variations in sample size, differences in the demographic composition of the study populations, geographic and environmental influences, sociocultural and economic conditions, the type of biological samples analyzed, the age distribution of participants, and variations in diagnostic methodologies.

Due to the evidence of heterogeneity across studies (I^2^ = 97.1% and p < 0.001), subgroup analysis was conducted by country, year of publications, study population and sample size. The result revealed that the highest pooled prevalence of leukemia was 10.58% in Nigeria, followed by 7.31% in Ethiopia, and the lowest was 0.58% in Zambia. The difference in leukemia prevalence between countries could be attributed to a combination of genetic, environmental, infectious, healthcare, and socioeconomic factors. Additionally, Nigeria has higher industrial activity (oil refining, mining, pesticides) compared to Zambia, increasing exposure to benzene, formaldehyde, and other leukemia-linked chemicals.

When stratified by publication year, studies published before 2015 reported a prevalence of 4.16%, whereas those published after 2016 reported a higher prevalence of 6.67%. The difference in prevalence could due to an increased awareness and improved diagnostic methods over time may have led to higher detection rates, while changes in environmental or lifestyle risk factors could have contributed to a true rise in cases. Additionally, variations in study design, sample selection, or diagnostic criteria between earlier and later research might explain the discrepancy. Alternatively, this may be attributed to improvements in leukemia diagnosis over time and better detection of cases.

Subgroup analysis by population revealed a 3.38% prevalence (95% CI: 1.68–5.08) in children and 7.15% (95% CI: 4.42–9.89) in the general population, indicating a significantly higher burden in the latter. Both estimates showed substantial heterogeneity (I^2^ > 95%, p < 0.001), reflecting notable variability across studies. The observed difference in prevalence could be attributed to several key factors. First, biological and developmental differences may play a role, as children’s immune systems, metabolic processes, or environmental exposures often differ from adults. Second, variations in risk factors such as lifestyle, comorbidities, or behavioral patterns could contribute to lower susceptibility in pediatric populations. Third, diagnostic and screening practices may differ, as children are less likely to undergo routine testing for certain conditions compared to adults. Additionally, underreporting or misclassification in pediatric studies could artificially reduce prevalence estimates. The high heterogeneity in both subgroups (I^2^ > 95%) further suggests methodological disparities, such as differences in study design, sampling, or diagnostic criteria.

Another subgroup analysis by sample size demonstrated notable variations in leukemia prevalence. Studies with smaller sample sizes (<384 participants) yielded a higher pooled estimate of 7.99%, though with considerable heterogeneity (I^2^ = 98.4%, p < 0.001). Conversely, studies with larger sample sizes (≥385 participants) showed a lower prevalence of 3.61%, with moderate heterogeneity (I^2^ = 89.4%, p < 0.001). The observed difference in leukemia prevalence between studies with smaller and larger sample sizes may be attributed to several methodological and clinical factors. Smaller studies often focus on high-risk or specialized populations, which could inflate prevalence estimates, whereas larger studies typically reflect more generalized populations, leading to lower but potentially more representative estimates. Additionally, smaller studies are more prone to random variability and selection bias due to limited sampling diversity, contributing to the substantial heterogeneity (I^2^ = 98.4%). In contrast, larger studies, despite moderate heterogeneity (I^2^ = 89.4%), benefit from broader participant inclusion and greater statistical power, enhancing reliability. Given that leukemia is a relatively rare disease, case ascertainment poses inherent challenges. These findings underscore the need for cautious interpretation of small-scale studies and emphasize the importance of large, population-based research to generate robust prevalence estimates.

Several key risk factors have been identified as significant predictors of leukemia. Among the factors older age and rural residency are significant predictors of leukemia in Africa. Pooled analysis revealed that individuals living in the rural area are 56.98 times more prone to leukemia than the urban residents (OR: 56.98, 95% CI: 9.97–326.70), and older age individuals are 3.90 times more likely to develop leukemia than their counter parts (OR: 3.90, 95% CI: 1.39–10.98). Advanced age is consistently associated with a higher risk, likely due to accumulated genetic mutations and declining immune function. Rural residency may contribute to increased exposure to environmental carcinogens, such as pesticides or industrial pollutants. Occupational and environmental exposures, including electromagnetic fields, electrical fields, and chemical substances, have also been linked to leukemia development. Additionally, metabolic conditions like diabetes mellitus (DM) and obesity may promote leukemogenesis through chronic inflammation and insulin resistance. Autoimmune diseases and HCV (hepatitis C virus) infection further elevate risk due to immune dysregulation and persistent viral effects. Socioeconomic factors, such as unemployment, may limit access to healthcare, delaying diagnosis and treatment of precancerous conditions. Together, these factors highlight the complex interplay of genetic, environmental, and lifestyle influences in leukemia risk [45].

This finding aligns with a systematic review and meta-analysis by Guo Y(45), in China [46], Khalade A [47] and a study in Ethiopia [26], they identified chemical substance exposure and HCV infection as a significant predictors of leukemia. Chemical exposure increases leukemia risk by damaging bone marrow DNA, causing mutations. Its metabolites disrupt blood cell production, leading to abnormal white blood cells. Chronic exposure can trigger myelodysplastic syndrome, progressing to acute myeloid leukemia (AML). Benzene also suppresses immune function, raising susceptibility to blood cancers. In contrast to studies from China [46]and Ethiopia [26] fail to identify obesity and DM as a predictor of leukemia, ours found association. The observed discrepancies in risk factor associations may stem from variations in sample size, study design, and the methodological approach to data pooling. Smaller sample sizes can reduce statistical power, potentially obscuring true associations or inflating spurious ones. Differences in study design such as case-control versus cohort studies may introduce selection bias or confounding effects.

Leukemia remains a major public health challenge in Africa, compounded by late diagnosis, limited treatment access, and inadequate diagnostic tools. Addressing this burden requires enhanced early detection, expanded access to advanced diagnostics such as flow cytometry, and affordable therapies. Strengthening healthcare infrastructure, alongside awareness campaigns and research, is essential for improving survival and patient outcomes.

### Strength and limitation

This study benefits from a comprehensive literature search and rigorous data evaluation. However, this review has several limitations. First, the limited number of studies meeting the inclusion criteria reduced the statistical power of our pooled analyses. Second, the exclusion of non-English publications due to a lack of translation resources may have introduced selection bias. Third, the predominance of studies reporting only incidence rates, with limited data on prevalence and risk factors, restricted our ability to conduct deeper etiological analyses. Fifth, this review includes literature published up to March 30, 2025. Studies published after that date were not included. Future updates may alter the conclusions. Additionally, some factors were reported by only a single article. Pooled odds ratios based on a single study may be unreliable and should be interpreted with caution.

## Conclusion

This meta-analysis estimates a 5.09% prevalence of leukemia in Africa, highlighting a significant disease burden. Older age and rural residency were identified as major risk factors, likely driven by healthcare disparities, environmental exposures, and diagnostic delays. Addressing this requires targeted interventions improved rural infrastructure, age-specific screening, and enhanced diagnostics alongside future longitudinal research to establish causality and region-specific strategies.

## Supporting information

S1 File Supplementary tables. This file contains **S1 table (**The search strategy and number of articles retrieved from the searched databases for the review prevalence and associated factors of leukemia in Africa.) and **S2 table (**Quality appraisal results of included articles**).**
(DOCX)

S2 File PRISMA 2020 Checklist. (DOCX)

## Abbreviations

ALL: acute lymphoblastic leukemia
AML: acute myeloid leukemia
CI: confidence interval
CLL: chronic lymphocytic leukemia
CML: chronic myeloid leukemia
HL: Hodgkin lymphoma
NHL: non-Hodgkin lymphoma.
