# Supplementary material for: Prevalence and associated factors of Leukemia in Africa: A systematic review and meta-analysis
Source: PLoS One. 2026 Aug 3;21(8):e0354814. doi: 10.1371/journal.pone.0354814 (PMC13432122; doi:10.1371/journal.pone.0354814)
Supplement: S1 File — This file contains S1 table (The search strategy and number of articles retrieved from the searched databases for the review prevalence and associated factors of leukemia in Africa.) and S2 table (Quality appraisal results of included articles). (DOCX) [file pone.0354814.s001.docx]

**Prevalence and associated factors of leukemia in Africa: A systematic review and meta-analysis**

**Supplementary files**

**List of tables**

[***S1 Table***: The search strategy and number of articles retrieved from the searched databases for the review prevalence and associated factors of leukemia in Africa. 1](#_Toc235381442)

[***S2 Table***: Quality appraisal results of included articles 2](#_Toc235381443)

S1 Table: The search strategy and number of articles retrieved from the searched databases for the review prevalence and associated factors of leukemia in Africa.

| Database | Searching strings | Number of paper identified |
| --- | --- | --- |
| PubMed | (((((((((((Prevalence) OR (Magnitude)) OR (Incidence)) AND (Predictors)) OR (factors)) OR (Risk-Factors)) OR (Associated factors)) AND (Leukemia)) OR (Hematological Malignancy)) OR (Cancer)) AND (Africa) by adding African search filter Filters: Free full text, Classical Article, Clinical Study, Clinical Trial, Randomized Controlled Trial, English, Humans, from 2000/1/1 - 2025/2/30 | 8156 |
| Science Direct | (prevalence) OR (magnitude) AND (associated factor) OR (risk factor) AND (leukemia) OR hematological malignancy) AND (Africa) | 3478 |
| Scopus | prevalence* OR incidence* AND Leukemia* OR hematological AND abnormality AND risk AND factors* OR predictors* AND * Africa* | 2357 |
| Google scholar |  | 3540 |
| Other |  | 15 |

S2 Table: Quality appraisal results of included articles

| **Author’s Name** | Was the sample frame appropriate to address the target population? | Were study participants sampled appropriately? | Was the sample size adequate? | Were the study subjects and the setting described in detail? | Was the data analysis conducted with sufficient coverage of the identified sample? | Were valid methods used for the identification of the condition? | Was the condition measured in a standard, reliable way for all participants? | Was there appropriate statistical analysis? | Was the response rate adequate, and if not, was the low response rate managed appropriately? | Quality score |
| --- | --- | --- | --- | --- | --- | --- | --- | --- | --- | --- |
| Endalamaw A, et al. | Yes | Yes | Yes | Yes | Yes | Yes | Yes | Yes | Yes | 8 |
| Mousay HY, et al. | Yes | Yes | Yes | Yes | Yes | Yes | Yes | Yes | Yes | 8 |
| Haouas H,et al. | Yes | Yes | Yes | Yes | Yes | Yes | Yes | Yes | Yes | 8 |
| Abuidres DO,et al. | Yes | Yes | Yes | Yes | Yes | Yes | Yes | Yes | Yes | 8 |
| Kassahun W,et al. | Yes | Yes | Yes | Yes | Yes | Yes | Yes | Yes | Yes | 8 |
| Ugwu NI, et al. | Yes | Yes | Yes | Yes | Yes | Yes | Yes | Yes | Yes | 8 |
| Hussein S, et al. | Yes | Yes | Yes | Yes | Yes | Yes | Yes | Yes | Yes | 8 |
| Enawgaw B,et al. | Yes | Yes | Yes | Yes | Yes | Yes | Yes | Yes | Yes | 8 |
| Maybin Kalubula, et al. | Yes | Yes | Yes | Yes | Yes | Yes | Yes | Yes | Yes | 8 |
| Robert N, et al. | Yes | Yes | Yes | Yes | Yes | Yes | Yes | Yes | Yes | 8 |
| Ebrahim H, et al. | Yes | Yes | Yes | Yes | Yes | Yes | Yes | Yes | Yes | 8 |
| Alamin AA, et al. | Yes | Yes | Yes | Yes | Yes | Yes | Yes | Yes | Yes | 8 |
| Kagu M, et al. | Yes | Yes | Yes | Yes | Yes | Yes | Yes | Yes | Yes | 8 |
| Woldu M, et al. | Yes | Yes | Yes | Yes | Yes | Yes | Yes | Yes | Yes | 8 |
| Babatunde T, et al. | Yes | Yes | Yes | Yes | Yes | Yes | Yes | Yes | Yes | 8 |
