# Supplementary material for: Prevalence and associated factors of Leukemia in Africa: A systematic review and meta-analysis
Source: PLoS One. 2026 Aug 3;21(8):e0354814. doi: 10.1371/journal.pone.0354814 (PMC13432122; doi:10.1371/journal.pone.0354814)
Supplement: Attachment — Submitted filename: Authors point by point Response to Reviewers..docx [file pone.0354814.s004.docx]

Response to Reviewers

Date : June 16, 2026

Manuscript ID: PONE-D-26-23321

Title: Prevalence and associated factors of leukemia in Africa, A systematic review and meta-analysis

Dear Dr. Baysal, (Academic Editor)

Thank you for the opportunity to revise our manuscript. We also sincerely thank the editor and the reviewers for their time, effort, and constructive feedback. We have addressed all comments point‑by‑point and believe the manuscript has been substantially improved as a result.

Below, editor and reviewer comments are in “**bold**” with our responses in plain text. All page/line numbers refer to the "Revised Manuscript with Track Changes" file. Additionally, line numbers are provided on a per‑page basis, corresponding to the location of each change in the revised manuscript. Furthermore, in response to the reviewers' request for language and typographical corrections, we have thoroughly revised the entire manuscript. These corrections have been made throughout the document and are not highlighted in color, as they are integrated comprehensively across the text.

Editor comment #1: **Please ensure that your manuscript meets PLOS ONE's style requirements, including those for file naming.**

Response: We have reformatted the manuscript accordingly. All files now follow PLOS ONE naming conventions.

Editor comment #2: **We note that the most recent literature search was completed over a year before the manuscript was submitted to the journal. Systematic and scoping reviews should provide an up-to-date overview of the research topic covered. Therefore, we request that you please update your searches as part of your revision to ensure that the most up-to-date literature is included in your review, or provide scientific justification for not doing so. Please ensure you mention this in your response to reviewers, and discuss whether this has any impact on the Results and Conclusions of the study. If you are submitting a protocol, it is only necessary to state whether the searches will be updated prior to the publication of the results.**

Response: We thank the editor for raising this important point. The original literature search was completed on March 30, 2025 and was comprehensive for its time. The delay between the search and submission was due to overlapping project demands and co‑author feedback timelines.

After careful consideration, we respectfully propose not to update the meta-analysis with newer studies for the following scientific reasons:

1. Our systematic review was designed to answer a specific research question based on literature available up to March 30, 2025. Incorporating post‑search studies would fundamentally change the scope and require a new review.

2. The purpose of our search was to ensure no relevant papers were missed during the original search period, which we are confident of, given our comprehensive search strategy.

To address the editor's concern transparently, we have added a limitation statement to the strength and limitation part (page 25, lines 17-18): "This review includes literature published up to March 30, 2025. Studies published after that date were not included. Future updates may alter the conclusions."

If the editor nonetheless requires an updated search, we are willing to undertake it, as we have sufficient time to complete the full re‑analysis.

Editor comment #3: **Please include a copy of Table 1 which you refer to in your text on page 14 in PDF submission.**

Response: We thank the editor for pointing this out. The table referenced as Table 1 on page 14 was presented with a heading that incorrectly labeled it as Table 2 on page 15. This typographical error has been corrected. The table heading now reads "Table 1," and all in-text citations have been aligned accordingly. Therefore, all in-text references and table headings throughout the manuscript have been corrected. (Page 11, 12, 14 and 19)

Editor comment #4: **We note you have included a table to which you do not refer in the text of your manuscript. Please ensure that you refer to Table 5 in your text; if accepted, production will need this reference to link the reader to the Table.**

Response: We appreciate the editor's insight. The issue was a typo error between the in‑text citation and the table heading. The table cited as Table 4 in the text had its heading incorrectly labeled as Table 5. There is no Table 5 in the manuscript. This typographical error has been corrected, and the table heading now reads Table 4, matching the in‑text citation. (Page 19)

Editor comment #5: **Please include captions for your Supporting Information files at the end of your manuscript, and update any in-text citations to match accordingly.**

Response: Thank you for this comment. We have added a "Supporting Information" section after the References with captions for all files. In-text citations have been updated to match the file names. The changes are visible on page 5, lines 21.and its caption description page 29 line 16-17 for **S1 Table**. **S1 file** is about the PRISMA 2020 Checklist, which is applicable for all the review process (page 29).

Editor comment #6: **If the reviewer comments include a recommendation to cite specific previously published works, please review and evaluate these publications to determine whether they are relevant and should be cited. There is no requirement to cite these works unless the editor has indicated otherwise.**

Response: We appreciate the editor's insight. To clarify: no reviewer asked us to cite any specific previously published work. However, one reviewer did recommend that we update older references in the introduction with more recent papers. We have revised the introduction accordingly (page 3 and 4).

**Review Comments to the Author**

**Reviewer 1 comments**

Review 1 comment #1: **more cautious interpretation of the pooled prevalence given extreme heterogeneity and publication bias,**

Response: We thank the reviewer for this important insight. We agree that the pooled prevalence should be interpreted cautiously given the extreme heterogeneity and evidence of publication bias. To address this, we have expanded the Discussion to explicitly caution against over interpreting the pooled estimate under these conditions. Furthermore, we have employed subgroup analysis to explore sources of heterogeneity and conducted a trim-and-fill analysis to adjust for publication bias. These additions are described in the Methods (page 11, lines 15-19) and Result section (page 13, lines 25-26).

Review 1 comment #2: **clearer reporting of predictor analyses and their limitations**

Response: We thank the reviewer for this helpful suggestion. We have now clarified the reporting of predictor analyses and their limitations. Specifically:

1. We have expanded the Methods section to describe how predictor variables were selected and analyzed (page 7, lines 7-8).
2. We have added a clear statement of limitations regarding the predictor analyses in the Discussion, including issues such as some of the factors are reported only by one author or low statistical power] (page 26, lines 19-20).

These revisions ensure that readers can properly interpret the predictor findings without overgeneralization.

Review 1 comment #3: **an explicit Data Availability statement with access to underlying extracted data/analysis materials.**

Response: We appreciate the reviewer's comment. We have added an explicit Data Availability statement indicating that all extracted data and analysis materials are provided as Supporting Information files (S1 checklist, and S1Table) and also the excel that the actual meta-analyses performed were available from the corresponding author upon reasonable request. The statement appears on (page 27, lines 16-18).

Review 1 comment #4: **language and formatting polishing.**

Response: We appreciate the reviewer's comment. The manuscript has been professionally polished for language and formatting according to PLOS ONE standards. Changes are evident throughout the revised document.

-------------------------------------------------------------------------------------------------------------

**Reviewer 2 comments**

Review 2 comment #1: **The abstract says that "meta-regression were conducted to assess heterogeneity". As I see it, meta-regression does not assess heterogeneity itself, but helps assessing possible sources of heterogeneity (specifically, it assess the possible influence of moderating variables).**

Response: We thank the reviewer for this important clarification. The reviewer is correct that meta-regression does not assess heterogeneity itself but rather helps identify possible sources of heterogeneity by examining the influence of moderating variables. We have revised the abstract and the main manuscript accordingly. The corrected sentence now reads: "meta-regression was conducted to explore potential sources of heterogeneity" (abstract, page 2, line 12; main text, page 20, line 5-6).

Review 2 comment #2: **The introduction cites global leukemia prevalence from 2015 and 2012. Perhaps more recent data could be better.**

Response: We thank the reviewer for this helpful suggestion. We agree that more recent data on global leukemia prevalence are preferable. Accordingly, we have replaced the outdated references with more current sources that illustrate the global and regional burden of leukemia. These changes have been made in the introduction (page 3, lines 2-32) and are highlighted in the revised manuscript.

Review 2 comment #3: **When describing the search strategy, the manuscript says that some databases were used, "along with relevant institutional repositories (SG and MAR)". Now, I assume SG and MAR were the researchers who made the searches, but the sentence in its current form suggests that SG and MAR are institutional repositories.**

Response: We thank the reviewer for this keen observation. The reviewer is correct that the original wording was confusing and grammatically incorrect, as it implied that "SG" and "MAR" were institutional repositories. In fact, SG and MAR are initials of the two reviewers who performed the database searches. We have revised the sentence to clarify this. The corrected text now states that the database searches were conducted independently by two reviewers (SG and MAR). This change has been made on (page 5, lines 8-10).

Review 2 comment #4: **The paragraph entitles "Outcomes of the study" is exposing the goals of the meta-analysis, which had already been described eariler in the Introduction. I would suggest deleting that paragraph.**

Response: We appreciate the reviewer's insightful observation. The paragraph was redundant, as the meta-analysis goals were already stated in the Introduction. We have deleted the paragraph as suggested.

Review 2 comment #5: **"Joanna Bridge Institute" should be "Joanna Briggs Institute"**.

Response: We thank the reviewer for catching this typographical error. "Joanna Bridge Institute" has been corrected to "Joanna Briggs Institute" (page 6, lines 12).

Review 2 comment #6: **Tables are mis numbered. Table 2 should be Table 1, Table 3 should de Table 2, and so forth. Actually, there is a sentence saying "...thorough evaluation and synthesis of the available evidence on leukemia epidemiology across African populations (Table 1)". I do not know whether this Table 1 is the currentl numbered Table 2 or another, now missing table.**

Response: We thank the reviewer for carefully identifying this typographical error. We agree that the tables were misnumbered. All tables have now been renumbered correctly: former Table 2 is now Table 1, former Table 3 is now Table 2, and so forth. All in-text citations have been updated accordingly. These corrections are visible throughout the revised manuscript.

Review 2 comment #7: **In Table 2 (in its current numbering), "libiya" should be "Libya".**

Response: We thank the reviewer for catching this typographical error. "Libiya" has been corrected to "Libya" in the relevant table (now correctly numbered as Table 1 after our renumbering; see page 11, line 4).

Review 2 comment #8: **When speaking about publication bias, the manuscript correctly says that publication bias "occurs when studies with statistically significant or favourable results are more likely to be published than those with null or negative findings". Now, in the context of this specific meta-analysis which analyzes prevalences, the idea of null or negative results translates into low (but not bengative, of course) prevalences. Perhaps the manuscript could directly apply the general concept of publication bias to the specific context of this particular meta-analysis.**

Response: We thank the reviewer for this insightful observation. The reviewer is correct that the general definition of publication bias (referring to "null or negative findings") does not directly translate to prevalence studies, where findings are proportions rather than directional effects. We have now revised the paragraph to specifically address publication bias in the context of prevalence studies, noting that studies reporting unusually high or low prevalence estimates may be more likely to be published, while studies reporting moderate or "non-significant" prevalence estimates may be underrepresented. This revision is located on page 11, lines 2-8. And page 13, lines 1-4.

Review 2 comment #9: **When analyzing publication bias, the manuscript reasonably concludes that studies with low sample sizes require larger prevalences to be published. However, I believe there is another possible explanation. Keeping in mind that the manuscript is analyzing prevalences from a relatively unfrequent pathology, it is possible that some of the low sample size studies simply did not detect cases of leukemia due to sampling variability, and thus they would not be included in the meta-analysis. In other words, studies with low sample sizes may contain either zero or a negligible number of patients with leukemia, and thus those studies are not relevant to this meta-analysis.**

Response: We thank the reviewer for this critical insight. The reviewer raises an excellent alternative explanation: for a less frequent pathology like leukemia, small sample size studies may detect zero or negligible cases due to sampling variability, rendering them ineligible for inclusion in the meta-analysis. This is an important nuance beyond the publication bias explanation. We have revised the Discussion to incorporate this point, acknowledging that both publication bias and the inherent difficulty of detecting rare events in small samples may contribute to the observed pattern.

Review 2 comment #10: **"p = 0.000" should be "p < 0.001)".**

Response: Thank you for this insight. "p = 0.000" has been corrected to "p < 0.001" throughout the manuscript.

Review 2 comment #11: **In Figure 5, several countries only have one study, anf thus the line "Subtotal (i-squared = .%, P = .)" remained without data. I would suggest removing those empty lines.**

Response: We thank the reviewer for this insight. In order to remove the empty lines, we initially assumed that merging the studies and grouping them by African region: specifically into South Africa, North Africa, West Africa, and East Africa would solve the issue. However, the problem persists, as West Africa and South Africa each have only one study. Because of this, we prefer to revert to the original subgrouping, which was conducted on a country-specific basis. Finally, the figure in question is not editable and therefore cannot be deleted independently. As a result, this issue has been acknowledged as a limitation in the revised manuscript.

Review 2 comment #12: **In Table 5 (or perhaps 4) the manuscript presents the results of meta-regression analyses using study year and sample size as moderators. Were they analized separately or together? I assume they were analyzed separately.**

Response: We thank the reviewer for this insight. Yes, the variables were analyzed as separate moderators. During the analyses, each was selected and examined independently.

Review 2 comment #13: **Also in Table 5, the result od the meta-regression with the sample size is a bit odd, because Egger's test (which is essentialy the same analysis) was in fact significant.**

Response: We thank the reviewer for this critical insight. The reported result was copied directly from the software output. We acknowledge that the lower and upper confidence limits are close to the null.

Review 2 comment #14: **Figure 9 shows, among others, the effect of older age and being male on leukemia prevalence. Complementarily, the manuscript may perform meta-regressions with mean age and perecentage of males as quantitative variable to more precisely analyse the effect and to include more studies in the meta-regression analysis.**

Response: Thank you for this critical insight. While this would be valuable, not all articles reported mean age, and the percentage of males to females was not systematically extracted. These variables could therefore not be analyzed as moderators.

Review 2 comment #15: **The sentence which end as "...lower prevalences than a systematic review and meta-analysis 53.69%(39), Pakistan(40), Bangladesh(41),and India(42)" may need some rephrasing.**

Response: We appreciate the reviewer's insight. Rephrasing has been made accordingly (page 22, 16-18).

Sincerely,

Solomon Gedfie (corresponding author), on behalf of co‑authors
