# Supplementary material for: Prevalence and associated factors of Leukemia in Africa: A systematic review and meta-analysis
Source: PLoS One. 2026 Aug 3;21(8):e0354814. doi: 10.1371/journal.pone.0354814 (PMC13432122; doi:10.1371/journal.pone.0354814)
Supplement: Peer review history

# Peer review: Decision Letter 0

**DOI:** 10.1371/journal.pone.0354814.r001

**Authors:** Mehmet Baysal

4 Jun 2026

PONE-D-26-23321

Prevalence and associated factors of leukemia in Africa, A systematic review and meta-analysis

PLOS One

Dear Dr. Gedfie,

Thank you for submitting your manuscript to PLOS ONE. After careful consideration, we feel that it has merit but does not fully meet PLOS ONE’s publication criteria as it currently stands. Therefore, we invite you to submit a revised version of the manuscript that addresses the points raised during the review process.

-->The study holds strong potential for publication, the high heterogeneity and publication bias require an interpretation. Please clarify your predictor analyses, add a Data Availability statement, and polish the formatting.

Please submit your revised manuscript by Aug 03 2026 11:59PM. If you will need more time than this to complete your revisions, please reply to this message or contact the journal office at plosone@plos.org. When you're ready to submit your revision, log on to https://www.editorialmanager.com/pone/ and select the 'Submissions Needing Revision' folder to locate your manuscript file.

Please include the following items when submitting your revised manuscript:

- A letter that responds to each point raised by the academic editor and reviewer(s). You should upload this letter as a separate file labeled 'Response to Reviewers'.
- A marked-up copy of your manuscript that highlights changes made to the original version. You should upload this as a separate file labeled 'Revised Manuscript with Track Changes'.
- An unmarked version of your revised paper without tracked changes. You should upload this as a separate file labeled 'Manuscript'.

If you would like to make changes to your financial disclosure, please include your updated statement in your cover letter. Guidelines for resubmitting your figure files are available below the reviewer comments at the end of this letter.

If applicable, we recommend that you deposit your laboratory protocols in protocols.io to enhance the reproducibility of your results. Protocols.io assigns your protocol its own identifier (DOI) so that it can be cited independently in the future. For instructions see: https://journals.plos.org/plosone/s/submission-guidelines#loc-laboratory-protocols. Additionally, PLOS ONE offers an option for publishing peer-reviewed Lab Protocol articles, which describe protocols hosted on protocols.io. Read more information on sharing protocols at https://plos.org/protocols?utm_medium=editorial-email&utm_source=authorletters&utm_campaign=protocols.

As the corresponding author, your ORCID iD is verified in the submission system and will appear in the published article. PLOS supports the use of ORCID, and we encourage all coauthors to register for an ORCID iD and use it as well. Please encourage your coauthors to verify their ORCID iD within the submission system before final acceptance, as unverified ORCID iDs will not appear in the published article. *Only*  the individual author can complete the verification step; PLOS staff *cannot*  verify ORCID iDs on behalf of authors.

We look forward to receiving your revised manuscript.

Kind regards,

Mehmet Baysal

Academic Editor

PLOS One

Journal Requirements:

When submitting your revision, we need you to address these additional requirements.

1. Please ensure that your manuscript meets PLOS ONE's style requirements, including those for file naming. The PLOS ONE style templates can be found at

https://journals.plos.org/plosone/s/file?id=wjVg/PLOSOne_formatting_sample_main_body.pdf and

https://journals.plos.org/plosone/s/file?id=ba62/PLOSOne_formatting_sample_title_authors_affiliations.pdf

2. We note that the most recent literature search was completed over a year before the manuscript was submitted to the journal. Systematic and scoping reviews should provide an up-to-date overview of the research topic covered. Therefore, we request that you please update your searches as part of your revision to ensure that the most up-to-date literature is included in your review, or provide scientific justification for not doing so. Please ensure you mention this in your response to reviewers, and discuss whether this has any impact on the Results and Conclusions of the study. If you are submitting a protocol, it is only necessary to state whether the searches will be updated prior to the publication of the results.

3. Please include a copy of Table 1 which you refer to in your text on page 14 in PDF submission.

4. We note you have included a table to which you do not refer in the text of your manuscript. Please ensure that you refer to Table 5 in your text; if accepted, production will need this reference to link the reader to the Table.

5.  Please include captions for your Supporting Information files at the end of your manuscript, and update any in-text citations to match accordingly. Please see our Supporting Information guidelines for more information: http://journals.plos.org/plosone/s/supporting-information.

6. If the reviewer comments include a recommendation to cite specific previously published works, please review and evaluate these publications to determine whether they are relevant and should be cited. There is no requirement to cite these works unless the editor has indicated otherwise.

[Note: HTML markup is below. Please do not edit.]

Reviewers' comments:

Reviewer's Responses to Questions

**Comments to the Author**

1. Is the manuscript technically sound, and do the data support the conclusions?

The manuscript must describe a technically sound piece of scientific research with data that supports the conclusions. Experiments must have been conducted rigorously, with appropriate controls, replication, and sample sizes. The conclusions must be drawn appropriately based on the data presented.

Reviewer #1: Partly

Reviewer #2: Yes

**********

2. Has the statistical analysis been performed appropriately and rigorously?

Reviewer #1: No

Reviewer #2: Yes

**********

3. Have the authors made all data underlying the findings in their manuscript fully available?

The PLOS Data policy requires authors to make all data underlying the findings described in their manuscript fully available without restriction, with rare exception (please refer to the Data Availability Statement in the manuscript PDF file). The data should be provided as part of the manuscript or its supporting information, or deposited to a public repository. For example, in addition to summary statistics, the data points behind means, medians and variance measures should be available. If there are restrictions on publicly sharing data—e.g. participant privacy or use of data from a third party—those must be specified.

Reviewer #1: No

Reviewer #2: Yes

**********

4. Is the manuscript presented in an intelligible fashion and written in standard English?

PLOS ONE does not copyedit accepted manuscripts, so the language in submitted articles must be clear, correct, and unambiguous. Any typographical or grammatical errors should be corrected at revision, so please note any specific errors here.

Reviewer #1: Yes

Reviewer #2: Yes

**********

5. Review Comments to the Author

Please use the space provided to explain your answers to the questions above. You may also include additional comments for the author, including concerns about dual publication, research ethics, or publication ethics. (Please upload your review as an attachment if it exceeds 20,000 characters)

Reviewer #1: The study is potentially publishable, but I recommend major revision focused on (1) more cautious interpretation of the pooled prevalence given extreme heterogeneity and publication bias, (2) clearer reporting of predictor analyses and their limitations, (3) an explicit Data Availability statement with access to underlying extracted data/analysis materials, and (4) language and formatting polishing.

Reviewer #2: Thank you for the opportunity to review this manuscript. The manuscript concists of a meta-analysis which studies the prevalence of leukemia across African countries and explores possible moderating factors. I have some comment which I hope will help improve the manuscript.

1. The abstract says that "meta-regression were conducted to assess heterogeneity". As I see it, meta-regression does not assess heterogeneity itself, but helps assessing possible sources of heterogeneity (specifically, it assess the possible influence of moderating variables).

2. The introduction cites global leukemia prevalence from 2015 and 2012. Perhaps more recent data could be better.

3. When describing the search strategy, the manuscript says that some databases were used, "along with relevant institutional repositories (SG and MAR)". Now, I assume SG and MAR were the researchers who made the searches, but the sentence in its current form suggests that SG and MAR are institutional repositories.

4. The paragraph entitles "Outcomes of the study" is exposing the goals of the meta-analysis, which had already been described eariler in the Introduction. I would suggest deleting that paragraph.

5. "Joanna Bridge Institute" should be "Joanna Briggs Institute".

6. Tables are mis numbered. Table 2 should be Table 1, Table 3 should de Table 2, and so forth. Actually, there is a sentence saying "...thorough evaluation and synthesis of the available evidence on leukemia epidemiology across African populations (Table 1)". I do not know whether this Table 1 is the currentl numbered Table 2 or another, now missing table.

7. In Table 2 (in its current numbering), "libiya" should be "Libya".

8. When speaking about publication bias, the manuscript correctly says that publication bias "occurs when studies with statistically significant or favourable results are more likely to be published than those with null or negative findings". Now, in the context of this specific meta-analysis which analyzes prevalences, the idea of null or negative results translates into low (but not bengative, of course) prevalences. Perhaps the manuscript could directly apply the general concept of publication bias to the specific context of this particular meta-analysis.

9. When analyzing publication bias, the manuscript reasonably concludes that studies with low sample sizes require larger prevalences to be published. However, I believe there is another possible explanation. Keeping in mind that the manuscript is analyzing prevalences from a relatively unfrequent pathology, it is possible that some of the low sample size studies simply did not detect cases of leukemia due to sampling variability, and thus they would not be included in the meta-analysis. In other words, studies with low sample sizes may contain either zero or a negligible number of patients with leukemia, and thus those studies are not relevant to this meta-analysis.

10. "p = 0.000" should be "p < 0.001)".

11. In Figure 5, several countries only have one study, anf thus the line "Subtotal (i-squared = .%, P = .)" remained without data. I would suggest removing those empty lines.

12. In Table 5 (or perhaps 4) the manuscript presents the results of meta-regression analyses using study year and sample size as moderators. Were they analized separately or together? I assume they were analyzed separately.

12 bis. Also in Table 5, the result od the meta-regression with the sample size is a bit odd, because Egger's test (which is essentialy the same analysis) was in fact significant.

13. Figure 9 shows, among others, the effect of older age and being male on leukemia prevalence. Complementarily, the manuscript may perform meta-regressions with mean age and perecentage of males as quantitative variable to more precisely analyse the effect and to include more studies in the meta-regression analysis.

14: The sentence which end as "...lower prevalences than a systematic review and meta-analysis 53.69%(39), Pakistan(40), Bangladesh(41),and India(42)" may need some rephrasing.

**********

6. PLOS authors have the option to publish the peer review history of their article (what does this mean?). If published, this will include your full peer review and any attached files.

If you choose “no”, your identity will remain anonymous but your review may still be made public.

**Do you want your identity to be public for this peer review?**  For information about this choice, including consent withdrawal, please see our Privacy Policy.

Reviewer #1: **Yes:** Ehsan Abedini

Reviewer #2: No

**********

[NOTE: If reviewer comments were submitted as an attachment file, they will be attached to this email and accessible via the submission site. Please log into your account, locate the manuscript record, and check for the action link "View Attachments". If this link does not appear, there are no attachment files.]

To ensure your figures meet our technical requirements, please review our figure guidelines: https://journals.plos.org/plosone/s/figures

You may also use PLOS’s free figure tool, NAAS, to help you prepare publication quality figures: https://journals.plos.org/plosone/s/figures#loc-tools-for-figure-preparation.

NAAS will assess whether your figures meet our technical requirements by comparing each figure against our figure specifications.

# Author comment: Author response to Decision Letter 1

**DOI:** 10.1371/journal.pone.0354814.r002

26 Jun 2026

Our detailed responses to the editor and reviewers are provided in a separate document labeled "Response to Reviewers."

Attachment Submitted filename: Authors point by point Response to Reviewers..docx

# Peer review: Decision Letter 1

**DOI:** 10.1371/journal.pone.0354814.r003

**Authors:** Mehmet Baysal

14 Jul 2026

Prevalence and associated factors of leukemia in Africa, A systematic review and meta-analysis

PONE-D-26-23321R1

Dear Dr. Gedfie,

We’re pleased to inform you that your manuscript has been judged scientifically suitable for publication and will be formally accepted for publication once it meets all outstanding technical requirements.

Within one week, you’ll receive an e-mail detailing the required amendments. When these have been addressed, you’ll receive a formal acceptance letter and your manuscript will be scheduled for publication.

An invoice will be generated when your article is formally accepted. Please note, if your institution has a publishing partnership with PLOS and your article meets the relevant criteria, all or part of your publication costs will be covered. Please make sure your user information is up-to-date by logging into Editorial Manager at Editorial Manager® and clicking the ‘Update My Information' link at the top of the page. For questions related to billing, please contact billing support.

If your institution or institutions have a press office, please notify them about your upcoming paper to help maximize its impact. If they’ll be preparing press materials, please inform our press team as soon as possible -- no later than 48 hours after receiving the formal acceptance. Your manuscript will remain under strict press embargo until 2 pm Eastern Time on the date of publication. For more information, please contact onepress@plos.org.

Kind regards,

Mehmet Baysal

Academic Editor

PLOS One

Additional Editor Comments (optional):

Reviewers' comments:

Reviewer's Responses to Questions

**Comments to the Author**

1. If the authors have adequately addressed your comments raised in a previous round of review and you feel that this manuscript is now acceptable for publication, you may indicate that here to bypass the “Comments to the Author” section, enter your conflict of interest statement in the “Confidential to Editor” section, and submit your "Accept" recommendation.

Reviewer #2: All comments have been addressed

**********

2. Is the manuscript technically sound, and do the data support the conclusions?

The manuscript must describe a technically sound piece of scientific research with data that supports the conclusions. Experiments must have been conducted rigorously, with appropriate controls, replication, and sample sizes. The conclusions must be drawn appropriately based on the data presented.

Reviewer #2: Yes

**********

3. Has the statistical analysis been performed appropriately and rigorously?

Reviewer #2: Yes

**********

4. Have the authors made all data underlying the findings in their manuscript fully available?

The PLOS Data policy requires authors to make all data underlying the findings described in their manuscript fully available without restriction, with rare exception (please refer to the Data Availability Statement in the manuscript PDF file). The data should be provided as part of the manuscript or its supporting information, or deposited to a public repository. For example, in addition to summary statistics, the data points behind means, medians and variance measures should be available. If there are restrictions on publicly sharing data—e.g. participant privacy or use of data from a third party—those must be specified.

Reviewer #2: Yes

**********

5. Is the manuscript presented in an intelligible fashion and written in standard English?

PLOS ONE does not copyedit accepted manuscripts, so the language in submitted articles must be clear, correct, and unambiguous. Any typographical or grammatical errors should be corrected at revision, so please note any specific errors here.

Reviewer #2: Yes

**********

6. Review Comments to the Author

Please use the space provided to explain your answers to the questions above. You may also include additional comments for the author, including concerns about dual publication, research ethics, or publication ethics. (Please upload your review as an attachment if it exceeds 20,000 characters)

Reviewer #2: (No Response)

**********

7. PLOS authors have the option to publish the peer review history of their article (what does this mean?). If published, this will include your full peer review and any attached files.

If you choose “no”, your identity will remain anonymous but your review may still be made public.

**Do you want your identity to be public for this peer review?**  For information about this choice, including consent withdrawal, please see our Privacy Policy.

Reviewer #2: No

**********

# Editor report: Acceptance letter

**DOI:** 10.1371/journal.pone.0354814.r004

**Authors:** Mehmet Baysal

PONE-D-26-23321R1

PLOS One

Dear Dr. Gedfie,

I'm pleased to inform you that your manuscript has been deemed suitable for publication in PLOS One. Congratulations! Your manuscript is now being handed over to our production team.

At this stage, our production department will prepare your paper for publication. This includes ensuring the following:

* All references, tables, and figures are properly cited

* All relevant supporting information is included in the manuscript submission,

* There are no issues that prevent the paper from being properly typeset

You will receive further instructions from the production team, including instructions on how to review your proof when it is ready. Please keep in mind that we are working through a large volume of accepted articles, so please give us a few days to review your paper and let you know the next and final steps.

Lastly, if your institution or institutions have a press office, please let them know about your upcoming paper now to help maximize its impact. If they'll be preparing press materials, please inform our press team within the next 48 hours. Your manuscript will remain under strict press embargo until 2 pm Eastern Time on the date of publication. For more information, please contact onepress@plos.org.

You will receive an invoice from PLOS for your publication fee after your manuscript has reached the completed accept phase. If you receive an email requesting payment before acceptance or for any other service, this may be a phishing scheme. Learn how to identify phishing emails and protect your accounts at https://explore.plos.org/phishing.

If we can help with anything else, please email us at customercare@plos.org.

Thank you for submitting your work to PLOS ONE and supporting open access.

Kind regards,

PLOS ONE Editorial Office Staff

on behalf of

Dr. Mehmet Baysal

Academic Editor

PLOS One
